# Consensus elements for observational research on COVID-19-related long-term outcomes

**DOI:** 10.1097/MD.0000000000031248

**Published:** 2022-11-18

**Authors:** Andrew J. Admon, Pandora L. Wander, Theodore J. Iwashyna, George N. Ioannou, Edward J. Boyko, Denise M. Hynes, C. Barrett Bowling, Amy S.B. Bohnert, Ann M. O’Hare, Valerie A. Smith, John Pura, Paul L. Hebert, Edwin S. Wong, Meike Niederhausen, Matthew L. Maciejewski

**Affiliations:** a VA Center for Clinical Management Research, LTC Charles Kettles VA Medical Center, Department of Internal Medicine, University of Michigan Medical School, Department of Epidemiology, University of Michigan School of Public Health, Ann Arbor, MI, USA; b Veterans Affairs Puget Sound Health Care System, Department of Medicine, University of Washington, Seattle, WA, USA; c Pulmonary and Critical Care Medicine, Department of Medicine, Johns Hopkins University, Health Policy and Management, Bloomberg School of Public Health, Johns Hopkins University, Baltimore, MD, VA Center for Clinical Management Research, LTC Charles Kettles VA Medical Center, Department of Internal Medicine, University of Michigan, Ann Arbor, Michigan; U-M Institute for Healthcare Policy and Innovation, Ann Arbor, MI, USA; d Divisions of Gastroenterology, Veterans Affairs Puget Sound Healthcare System and University of Washington, Research and Development, Veterans Affairs Puget Sound Health Care System, Seattle Epidemiologic Research and Information Center, Seattle, WA, USA; e Veterans Affairs Puget Sound Health Care System Seattle Division, Seattle, Washington; Department of Medicine, University of Washington, Seattle, WA, USA; f Center to Improve Veteran Involvement in Care, VA Portland health care System, Portland, OR, College of Public Health and Human Sciences, and Center for Quantitative Life Sciences, Oregon State University, Corvallis, OR, USA; g Durham Veterans Affairs Geriatric Research Education and Clinical Center, Durham Veterans Affairs Medical Center (VAMC), Department of Medicine, Duke University, Durham, NC, USA; h VA Center for Clinical Management Research, LTC Charles Kettles VA Medical Center, Department of Anesthesiology, University of Michigan Medical School, Department of Epidemiology, University of Michigan School of Public Health, U-M Institute for Healthcare Policy and Innovation, Ann Arbor, MI, USA; i Hospital and Specialty Medicine Service and Seattle-Denver Center of Innovation, VA Puget Sound Health Care System and Department of Medicine, University of Washington, Seattle, WA, USA; j Center of Innovation to Accelerate Discovery and Practice Transformation (ADAPT), Durham Veterans Affairs Health Care System, Department of Population Health Sciences, Duke University Medical Center, Division of General Internal Medicine, Department of Medicine, Duke University, Durham, NC, USA; k Department of Biostatistics and Bioinformatics, Duke University, Durham, NC, USA; l Veterans Affairs Puget Sound Health Care System, Department of Medicine, University of Washington School of Public Health, Seattle WA, USA; m Center for Veteran-Centered and Value-Driven Care, VA Puget Sound Health Care System; Department of Health Services, University of Washington, Seattle, WA, USA; n Center to Improve Veteran Involvement in Care, VA Portland health care System, Oregon Health and Science University-Portland State University School of Public Health, Oregon Health and Science University, Portland, OR, USA; o Center of Innovation to Accelerate Discovery and Practice Transformation, Durham VA Medical Center; Department of Population Health Sciences, Duke University, Division of General Internal Medicine, Department of Medicine, Duke University, Durham, NC, USA.

**Keywords:** COVID-19, epidemiology, mortality, observational research, outcomes research, veterans

## Abstract

Severe acute respiratory syndrome coronavirus 2 (SARS-CoV-2) infection and its long-term outcomes may be jointly caused by a wide range of clinical, social, and economic characteristics. Studies aiming to identify mechanisms for SARS-CoV-2 morbidity and mortality must measure and account for these characteristics to arrive at unbiased, accurate conclusions. We sought to inform the design, measurement, and analysis of longitudinal studies of long-term outcomes among people infected with SARS-CoV-2. We fielded a survey to an interprofessional group of clinicians and scientists to identify factors associated with SARS-CoV-2 infection and subsequent outcomes. Using an iterative process, we refined the resulting list of factors into a consensus causal diagram relating infection and 12-month mortality. Finally, we operationalized concepts from the causal diagram into minimally sufficient adjustment sets using common medical record data elements. Total 31 investigators identified 49 potential risk factors for and 72 potential consequences of SARS-CoV-2 infection. Risk factors for infection with SARS-CoV-2 were grouped into five domains: demographics, physical health, mental health, personal social, and economic factors, and external social and economic factors. Consequences of coronavirus disease 2019 (COVID-19) were grouped into clinical consequences, social consequences, and economic consequences. Risk factors for SARS-CoV-2 infection were developed into a consensus directed acyclic graph for mortality that included two minimally sufficient adjustment sets. We present a collectively developed and iteratively refined list of data elements for observational research in SARS-CoV-2 infection and disease. By accounting for these elements, studies aimed at identifying causal pathways for long-term outcomes of SARS-CoV-2 infection can be made more informative.

## 1. Introduction

Severe acute respiratory syndrome coronavirus 2 (SARS-CoV-2) has infected nearly 80 million Americans through March, 2022^[1]^ and 500 million worldwide. Recovery is incomplete—or new sequelae arise—in up to half of adults after their initial infection.^[2–5]^ This crisis has led to extraordinary efforts to characterize risk factors and outcomes of coronavirus disease 2019 (COVID-19) and develop interventions for the post-acute sequalae of COVID-19 (PASC), also known as “long COVID” or “post COVID-19 conditions.”^[6,7]^

Such efforts are essential to the effective guidance and support of patients, caregivers, clinicians, policymakers, and all others affected by the pandemic. Yet, the complex array of biomedical, social, and environmental factors contributing to SARS-CoV-2 testing, infection, and outcomes poses challenges to researchers seeking to identify causal pathways to COVID-19 sequelae. Analytical approaches guided by causal diagrams may enable unbiased and accurate inferences despite such complex causal structures.^[8]^ In order to apply these methods, investigators must be able to understand the broad set of factors involved with SARS-CoV-2 infection and articulate how these factors relate to the risk of SARS-CoV-2 infection, to patient outcomes, and how risk factors relate to one another. Once these relationships are identified in a causal diagram, measurements of these factors can be undertaken to design effective studies focused on casual risk factor identification, treatment effect estimation, and mediation analysis.

To inform the design, measurement, and analysis of longitudinal studies of long-term outcomes of persons infected with SARS-CoV-2, we iteratively developed and refined a list of clinical, social, and economic factors hypothesized to be involved in SARS-CoV-2 infection and clinical outcomes. To accomplish this, we drew from both emerging insights into COVID-19 epidemiology and the knowledge of a national group of experts in the study of inpatient and ambulatory care, mental health, post-acute care, the social determinants of health, and long-term clinical outcomes after acute illnesses.

In this manuscript, we present the resulting list of data elements collectively identified to be relevant to a broad set of analyses seeking to understand the causal effects of SARS-CoV-2 infection. Additionally, we provide a consensus causal diagram specific to COVID-19-related long-term mortality. Our goal in developing the causal diagram was to guide nascent conceptual, measurement, and analytical efforts; as our collective knowledge of COVID-19 epidemiology expands, applying these findings in refined causal models will facilitate the robust identification of causal relationships that could potentially be targeted in efforts to improve care.

## 2. Methods

### 2.1. Veterans Health Administration COVID-19 Observational Research Collaboratory

The Veterans Health Administration’s COVID-19 Observational Research Collaboratory (CORC) was funded by the Department of Veterans Affairs (VA) Office of Research and Development to study long-term outcomes after COVID-19 infecton (see https://www.research.va.gov/corc/). Given the nascent evidence base related to COVID-19, CORC included the expertise of a national group of investigators with clinical and research expertise in inpatient and ambulatory health service provision, acute illness care, epidemiology, and long-term social, economic, and clinical outcomes. The CORC methods subgroup was tasked with identifying data elements necessary to support robust observational studies of COVID-19 epidemiology. This included the development of a consensus causal diagram to identify potential problems with confounding and selection bias; these biases were selected because they are readily identified on causal diagrams and, when identified at the outset of a study, can be avoided through data collection and analytical decisions.^[8–11]^ While the study team was VA-based and was focused on outcomes of Veterans with COVID-19 infection, the group’s intention was to provide a framework for causal research on COVID-19 among both Veterans and non-Veterans. CORC and its overarching data collection has been approved by the institutional review boards at the Ann Arbor, Durham, Portland, and Seattle VAs. This CORC sub-study involved minimal risk to the expert participants, did not link participant information to survey or DAG group responses, and did not offer compensation for participation.

### 2.2. Definitions

#### 2.2.1. Causal diagrams.

Causal diagrams are commonly used to illustrate relationships among risk factors and outcomes in health-related research.^[8,12]^ Such diagrams can guide the analysis and interpretation of clinical data by articulating sources of confounding and common biases, such as selection bias.^[10,13,14]^ If available prior to data collection, causal diagrams can help make analytic efforts maximally informative by enabling the measurement of data elements necessary for the accurate identification of exposure-outcome relationships and mediating pathways, and identifying variables that do not need separate measurement.

**Figure 1.**
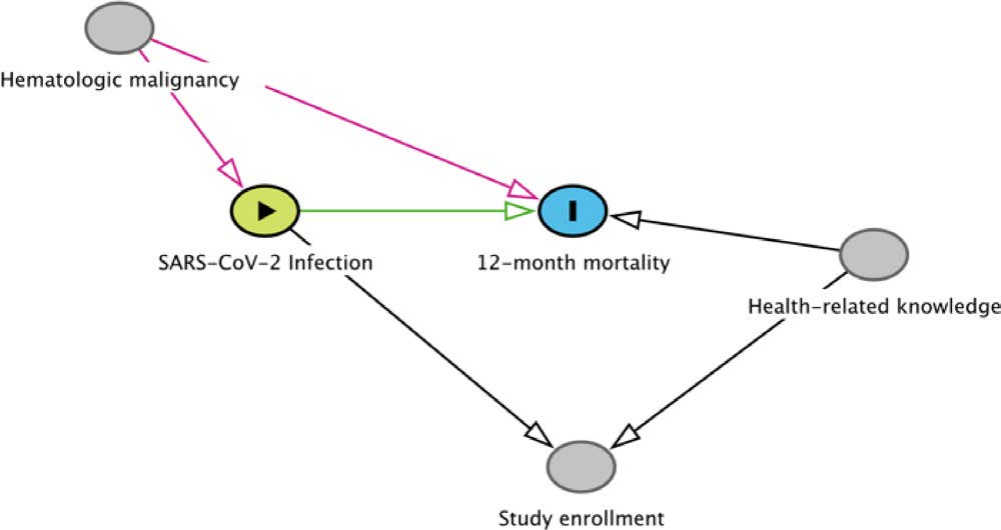
Directed acyclic graph (DAG) for a hypothetical study evaluating the impact of SARS-CoV-2 infection on 12-month mortality. SARS-CoV-2 = severe acute respiratory syndrome coronavirus 2.

Directed acyclic graphs (DAGs) are a type of causal diagram (Fig. 1).^[9]^ DAGs abide by several specific rules: variables (e.g., risk factors, exposures, and outcomes) are depicted as nodes; paths ending in arrowheads depict causal relationships and their directions (hence “directed”); and variables cannot cause themselves, either directly or through other variables (hence, the graphs are “acyclic”).^[15]^ Variables and their positions on a DAG can be informed by prior data, content knowledge, conceptual models, or assumptions, and inform analytical strategies to identify potentially causal relationships. By illustrating relationships among variables in an analysis, a DAG enables investigators to distinguish potential causal relationships from spurious or distorted ones (e.g., those due to confounding). Causal relationships so identified can inform development of preventive strategies, prognosis assessment, and potential therapeutic approaches.

The *minimally sufficient adjustment set* is the list of DAG elements that require adjustment (e.g., using regression, matching, or weighting) in order to accurately estimate the magnitude of the relationship between an exposure and an outcome.^[16]^ Minimum sufficient adjustment sets can be identified visually or by using DAG software.^[17]^ To identify the minimally sufficient adjustment set, two types of bias (omitted variable bias, collider bias) of potential risk factors must be articulated in the DAG.

### 2.3. Omitted variable bias and collider bias

Confounder variables explain variation in both the exposure and outcome variables, either directly or through intermediaries.^[8]^ Omitted variable bias is the bias stemming from confounder variables that are not accounted for the in the analysis. Consider a hypothetical study (Fig. 1) in which the investigators seek to measure the rate of 12-month mortality due to COVID-19 infection. Suppose that a hematologic malignancy increases the risk of a COVID-19 infection (the exposure) by lowering an individual’s innate immunity and increases the risk of 12-month mortality (the outcome) regardless of whether a patient develops COVID-19. An analysis that fails to measure and account for presence of hematologic malignancies as a confounder would likely result in an inflated estimate of 12-month mortality attributed to COVID-19. Other variables associated with both COVID-19 infection and 12-month mortality might similarly bias the estimated effect of COVID-19 on mortality. Whether the bias is away from or towards the null (i.e., no effect of the exposure on the outcome) depends on the direction and strength of their associations with both the exposure and the outcome.^[16,17]^ Sources of selection bias are often depicted on causal diagrams as *collider* variables.^[10,18,19]^ Suppose patients in the hypothetical study above are included in the study dataset after agreeing to enroll in the study (Fig. 1). Since the presence of COVID-19 infection and health-related knowledge may increase a person’s motivation to enroll in clinical research, both are likely (at least partial) determinants of study enrollment. In this case, “Study enrollment” functions as a *collider*, so-called because the arrowheads from “COVID-19 Infection” and “Health-related knowledge” meet, or collide, at this variable. In our hypothetical primary analysis that only includes patients who *enroll* in the study, selection bias due to this collider may result in a biased estimate of 12-month mortality attributed to COVID-19 infection. These biased estimates may not reflect the relationship that would be observed in a more inclusive population that did not depend on study enrollment. Strategies exist to help quantify or mitigate such selection bias due to selecting on a collider variable, but again require knowledge of the causal diagram and sufficient data to model the included variables and their relationships.^[20–22]^

### 2.4. Consensus identification of risk factors related to SARS-CoV-2 infection or outcomes

In March 2021, the CORC team developed a 10-item survey that was fielded to study investigators by email to solicit a list of candidate data elements related to SARS-CoV-2 infection or outcomes of infected patients. Study investigators compiled the list and grouped them into common domains. Candidate data elements were categorized as being either potentially causative of SARS-CoV-2 infection and testing (and so possible confounders) or consequent to SARS-CoV-2 infection (and so potentially important as outcomes or their mediators). Upon arriving at this set of candidate elements, four working groups composed of 50 total participants were convened via conference calls over two days to refine these elements and develop lists tailored to each of four specific outcomes: COVID-19 mortality, rehospitalization, behavioral and mental health, and financial consequences. Members of the DAG group were selected to maximize diversity in participant backgrounds, disciplines, and focus areas. Members of the DAG group included physicians, psychologists, nurse-scientists, social scientists, and public health researchers (see Table 1, http://links.lww.com/MD/H677, Supplemental Content, which lists the members of the DAG group and their expertise). Physicians represented primary care fields and specialties with experience treating COVID-19 and its complications. Scientific areas included infectious disease epidemiology, chronic disease epidemiology, health disparities, health services research, health policy, clinical epidemiology, and biostatistics. Each working group met, deliberated the candidate list of elements, and added elements specific to their assigned domain. Working groups were advised to be in their consideration of factors related to SARS-CoV-2 infection and outcomes among both Veterans and non-Veterans when developing each DAG so as to limit the risk of omitted variable bias (see Definitions, Omitted variable bias and collider bias).

**Table 1:** Unique COVID-19-related risk factors identified from 31 investigators.

Demographics	Physical health	Mental health	Personal social & economic factors	External social & economic Factors
Age	Asthma	Cognitive impairment	Access to PPE	Area group activities
Race/ethnicity	Blood type	Mental health diagnosis	Commute and most common form transport	Area spreader events
Sex	Body mass index	Stress	Congregate housing (e.g., nursing home)	Local adherence to public health guidelines
	Chronic obstructive pulmonary disease		Dining and grocery practices	Local and state policies
	Chronic kidney disease		Education	Proportion of friends, family, and other close contacts infected
	Coronary artery disease		Exposure to child care facilities	Urbanicity/rurality
	Diabetes		Front-line occupation	
	Diet		Health literacy	
	Frailty		Housing instability	
	Heart failure		How often need to leave home (e.g., for work, school, or other requirements)	
	Hypertension		Income	
	Immunodeficiency		Personal risk perception	
	Immunosuppressing medication		Social support	
	Lung cancer		Use of PPE	
	Other chronic lung diseases			
	Other chronic heart disease			
	Other cancer			
	Pregnancy			
	Recent surgery			
	Sickle cell disease			
	Sleep Disorders			
	Smoking			
	Total condition count			

COVID-19 = coronavirus disease 2019, PPE = personal protective equipment.

### 2.5. Directed acyclic graph development and operationalization

None of the four groups finalized the DAGs at the end of the 2-day remote meeting, so a core investigator group (AA, ASB, LW, MLM) convened over 4 remote meetings to finalize a candidate causal DAG specific to SARS-CoV-2 infection and 12-month mortality using data elements returned by the survey and group refinement process. The focus of the DAG was 12-month mortality because it was the primary outcome for several planned studies. To develop the DAG, the investigator group first grouped related elements (e.g., chronic heart disease and chronic kidney disease) into broader categories (e.g., comorbidities). This was done to improve readability of the resulting DAG while maintaining flexibility about specific variable modeling decisions later on. Next, the investigators confirmed that each included element was related to both SARS-CoV-2 infection and 12-month mortality. Finally, the investigators added candidate elements from emerging epidemiological studies published after the initial survey was written and distributed. Once drafted, this DAG was submitted to the broader investigator group for feedback and modification until a consensus was reached. Upon arriving at a finalized consensus DAG, the minimally sufficient adjustment sets of variables were identified. Finally, attention was paid to how DAG elements in the minimally sufficient adjustment set could be operationalized in standard electronic medical record data and supplemental datasets; this was done to support investigators seeking to immediately apply the DAG using available data.

## 3. Results

### 3.1. Potential causes and consequences of COVID-19 infection

31 investigators (of 65 initially contacted) responded to the email survey and contributed 49 distinct elements that could be characterized as potential risk factors for SARS-CoV-2 infection or determinants of clinical outcomes (Table 1). These included factors in five domains: demographic characteristics (age, ethnicity, race, and sex), physical health (e.g., body mass index, chronic lung diseases, diabetes), mental health (e.g., cognitive impairment, mental health diagnoses), personal social and economic factors (e.g., congregate housing, housing instability, and social support), and external social and economic factors (e.g., local adherence to public health guidelines, local and state policies, and urbanicity/rurality).

Respondents also suggested 72 potential clinical, social and economic consequences (or outcomes) of SARS-CoV-2 infection (Table 2). The clinical domain was divided into four subdomains: respiratory (e.g., chronic lung disease, hypoxemia), cardiometabolic (e.g., arrhythmia, thromboembolism, weight loss or gain), mental health and cognitive functioning (e.g., alcohol or substance use, anxiety, brain fog), and physical function (e.g., frailty, functional dependence, loss of smell or taste). The social domain was divided into three subdomains: relationship (e.g., guilt, isolation, stigma from infection), caregiving (e.g., childcare responsibilities, homeschooling, new pet ownership), and community/environmental factors (e.g., racism, food supplies, loss of family and friends, changing work culture). The potential economic consequences of SARS-CoV-2 infection were grouped into a health care and coverage domain (e.g., uninsurance/insurance disruption, health care access) and a personal finance domain (e.g., loss of income, wealth, home, or job).

**Table 2:** COVID-19-related clinical, social and economic outcomes identified from 31 investigators.

Clinical				Social			Economic	
Respiratory	Cardiometabolic	Mental Health and cognitive functioning	Other physical health and functioning	Relationship	Caregiving	Community/environmental	Health care and coverage	Personal finance
Chronic lung disease	Arrhythmia	Alcohol use	Auto-immune disorders	Divorce	Caregiving responsibilities for older adults	Automobile Collisions	Health care access	Childcare costs
Cough	Diabetes	Anhedonia	Coagulation disorders	Domestic violence	Challenges related to the education of children	Changing work culture	Health care use	Food assistance
Hypoxemia	Heart failure	Anxiety	Death	Guilt	Child care responsibilities	Changing social functions	Uninsurance or insurance disruption	Foregone income due to caregiving requirements
Obstructive sleep apnea	Hypertension	Brain fog	Frailty	Isolation	Homeschooling	Fear of public spaces		Health care spending
Smoking (e.g., cessation)	Other cardiovascular disease	Depression	Functional dependence (e.g., ADL and IADL impairment)	Loneliness	New pet ownership	Food bank availability and supply		Home loss
	Renal dysfunction	Dementia	Liver dysfunction	Stigma from infection		Loss of family or friends		Income loss
	Thromboembolism	Fatigue, stamina	Loss of smell or taste			Racism		Job loss or change
	Weight loss or gain	Post-traumatic stress disorder	Myopathy			Residence in a “food desert”		Loss of professional development activities
		Substance use	Muscle weakness			Satisfaction with health care		Wealth loss
		Suicide attempt	Neuropathy			Trust in public health		
			Other central nervous system disorders					
			Recurrent SARS-CoV-2 infection					
			Treatment or ventilator-related complications					

ADL = activity of daily living, COVID-19 = coronavirus disease 2019, IADL = instrumental activity of daily living, SARS-CoV-2 = severe acute respiratory syndrome coronavirus 2, ZIP = zone improvement plan.

### 3.2. Directed acyclic graph for mortality attributable to COVID-19 infection

**Figure 2.**
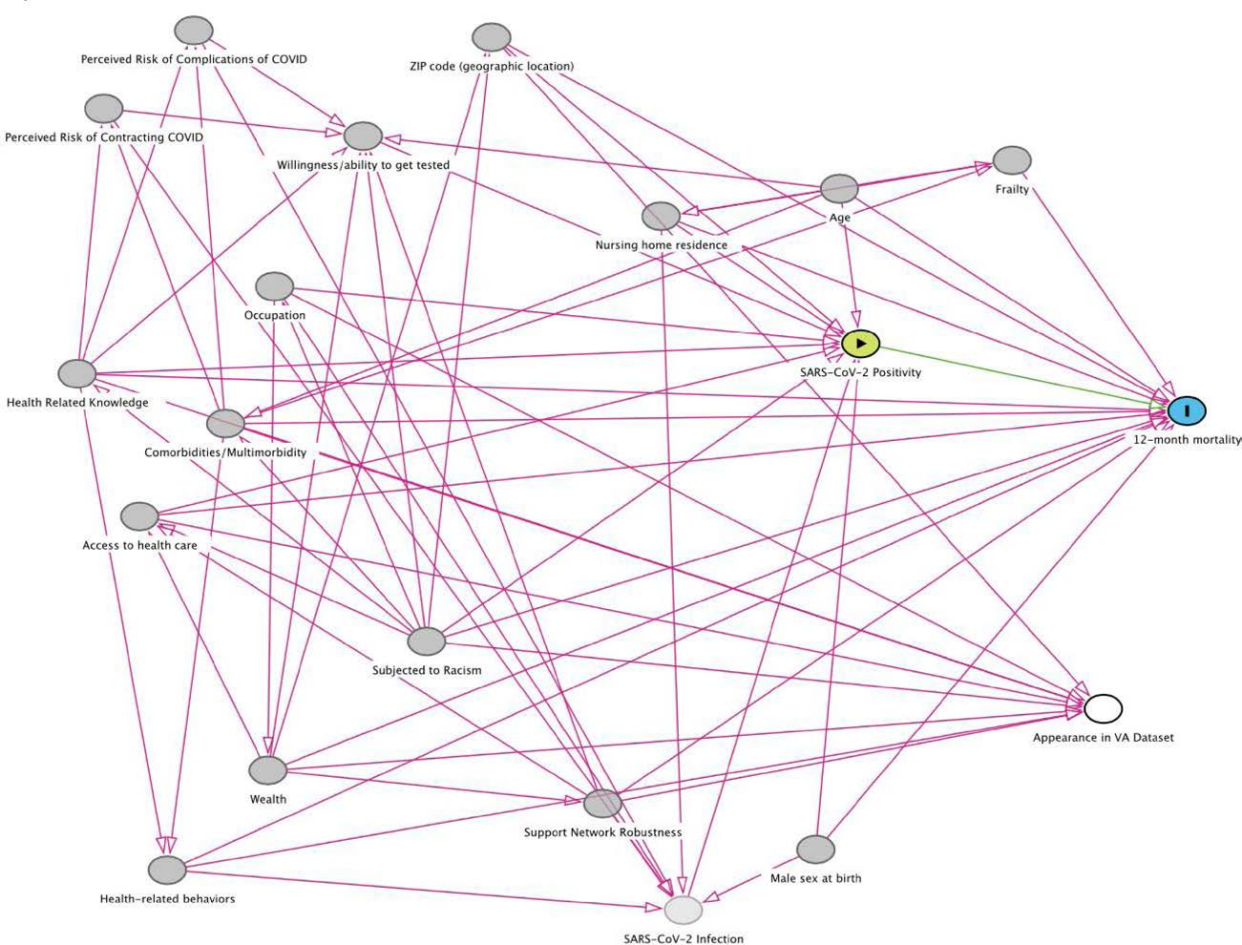
Consensus DAG describing the relationship between SARS-CoV-2 test positivity and 12-month mortality. DAG = directed acyclic graph; SARS-CoV-2 = severe acute respiratory syndrome coronavirus 2.

All characteristics appearing in Table 1 were candidates for inclusion in the DAG that characterized the effect of SARS-CoV-2 infection on mortality. For simplicity, candidate mediators (e.g., those identified in Table 2) were not included in the DAG for mortality. The consensus DAG for 12-month mortality, after iteration and approval by the entire group, is presented in Figure 2 (For Dagitty code, see Text 1, http://links.lww.com/MD/H678, Supplemental Content, which details Daggity code).^[17]^ The DAG includes 18 individual covariates related to a primary exposure of SARS-CoV-2 Positivity and 12-month mortality. Among these, “Appearance in VA Dataset” is modeled as a selection node to evaluate for any sources of collider stratification bias stemming from our sample construction using VA data.

The resulting DAG includes two minimally sufficient adjustment sets (see Text 2, http://links.lww.com/MD/H679, Supplemental Content, which details the variables in each adjustment set). Most of the variables in the two adjustment sets overlap. Many of the risk factors in these adjustment sets are individual-level variables available in VA data, including age, sex, race, ethnicity, marital status, comorbidity (via diagnoses and/or medications), zip code, and nursing home residence) or indices (e.g., frailty casemix measures) derived from individual-level variables. Notably, the variables not shared by the two sets included occupation, perceived risk of complications of COVID, perceived risk of contracting COVID, willingness/ability to get tested (set 1), and wealth (set 2).

Finally, the core investigator team identified strategies to operationalize any variable appearing in a minimally sufficient adjustment set, which is straightforward if based on a longstanding individual-level variable (see Table 3 for candidate measurement strategies). When specific variables were not readily available in VA electronic health record data, individual-level surrogates (e.g., receipt of applicable routine preventive care to proxy health-related behaviors) or aggregate surrogates (e.g., Medicaid eligibility to proxy wealth) based on health-related research were identified.^[23–25]^

**Table 3:** Operationalizing DAG concepts using common electronic health record data.

DAG concept	Proposed operationalization	References
Access to health care	Area deprivation index and area health resources	^[27,28]^
Age	Age in years	
Comorbidities/multimorbidity	Individual comorbidities and all combinations of 3 comorbidities; Charlson comorbidity index	^[29]^
Frailty	Risk analysis index or claims-based frailty index	^[30,31]^
Health-related behaviors	Receipt of applicable routine preventive care including annual wellness visits, indicated screening, and vaccinations; “Proportion of Days Covered” for chronic medications	^[23–25]^
Health-related knowledge	Receipt of applicable routine preventive care including annual vaccinations	^[23–25]^
Male sex at birth	Sex data	
Nursing home residence	Active community living center admission or Medicare claims for skilled nursing care	
Occupation	ZIP Code Median Income or Medicaid Eligibility	
Perceived risk of complications of COVID-19	Receipt of applicable routine preventive care including annual wellness visits, indicated screening, and vaccinations	^[23–25]^
Perceived risk of contracting COVID-19	Receipt of applicable routine preventive care including annual wellness visits, indicated screening, and vaccinations	^[23–25]^
Subjected to racism	Race and ethnicity data	
Support network robustness	Marital Status	
Wealth	ZIP code median income or medicaid eligibility	^[32]^
Willingness/ability to get tested	Drive time to nearest facility based on home ZIP code; Visit attendance frequency	^[33,34]^
ZIP code	Home ZIP code	

COVID-19 = coronavirus disease 2019, ZIP = zone improvement plan.

## 4. Discussion

In this manuscript, we describe the results of a consensus process to identify elements involved in both SARS-CoV-2 infection and long-term outcomes. Drawing on the expertise of a diverse group of clinicians and scientists, we developed, and iteratively refined lists of factors associated with SARS-CoV-2 infection and potential clinical, social, and economic outcomes. The resulting lists were developed into a causal diagram for long-term mortality to enable the robust identification of specific exposure-outcome relationships and mediating pathways. Finally, we operationalize specific DAG concepts into measurable covariates using electronic health record data. In so doing, we hope to support emerging scientific efforts focused on the long-term social, economic, and health-related outcomes of COVID-19.^[2,7,26]^

The exercise we describe offers a number of important insights that may be helpful in guiding future research efforts. First, both SARS-CoV-2 infection (unobserved) and SARS-CoV-2 test positivity (observed) are socially determined via factors that include occupation, support network robustness, health care access, and health-related knowledge. Studies that are limited to clinical data are unlikely to arrive at accurate measurements of the relationship between SARS-CoV-2 positivity and mortality. Second, determinants of SARS-CoV-2 infection include both health-related and social factors known to be robustly associated with a wide range of clinical outcomes (e.g., age, insurance status, diabetes). We also identified other characteristics that may be more specific to SARS-CoV-2 infection and resulting outcomes (e.g., residing in a long-term nursing home, dominant forms of transportation, adherence to public health guidance, childcare practices, etc.). Due to both disease- and transmission-specific features of SARS-CoV-2, the importance of these features in predicting infection, testing, and recovery may differ from prior epidemics and pandemics (e.g., HIV/AIDS, SARS-CoV-1, and pandemic influenza). As a result, existing case record forms or other data collection efforts using off-the-shelf instruments or previous analytical approaches might miss these important factors. Finally, our process yielded a number of specific clinical, social, and economic outcomes that may mediate some of the observed long-term consequences of COVID-19 (e.g., hypoxemia, anxiety, income loss); that could provide a starting point for causal mediation analyses that identify actionable pathways to 12-month mortality after COVID-19.

Our consensus DAG offers several additional insights. First, appearance in the VA dataset is *not* a source of selection bias as long as there is sufficient adjustment for variables in the minimally sufficient adjustment sets.^[19]^ Second, there are several minimally sufficient adjustment sets; certain characteristics that are likely difficult to accurately capture retrospectively (e.g., ‘perceived risk of contracting COVID-19 and perceived risk of complications of COVID-19) did not appear in each set, rendering it more likely to arrive at causal estimates of the impact of SARS-CoV-2 test positivity on 12-month mortality using available data or valid surrogates. Mediation analyses that share confounder sets are similarly estimable using available methods for causal mediation analysis.

Our process involved structured input from an interprofessional group of clinicians and scientists with a broad knowledge base and diverse expertise. As a result, identified risk factors include demographic data, physical and mental health factors, and both individual and external social, political, and economic factors. Importantly, survey participants were asked to submit risk factor nominations without consideration of measurability. When validated measures for consensus elements do not exist (e.g., personal protective equipment access and use), we expect that future efforts to develop them will prove helpful in attempts to measure causal effects. Similarly, our data collection effort yielded a broad range of clinical, social, and economic outcomes. By supporting the robust identification of COVID-19’s consequences across multiple domains, we hope to better position future work to alleviate its manifold effects.

Our study has the following potential limitations. First, the survey took place in early 2021 prior to the widespread availability of SARS-CoV-2 vaccination. We expect that many of the determinants of COVID-19 vaccination (e.g., health-related behaviors, perceived risk of contracting or complications of COVID-19, etc.) are already represented on the DAG; nevertheless, studies including both vaccinated and unvaccinated participants should additionally consider the determinants of vaccination that may differ from the determinants of SARS-CoV-2 infection and how they may be related COVID-19 outcomes. Second, both the survey findings and resulting DAG represent expert opinion based on evolving knowledge of COVID-19 epidemiology. While we purposely solicited opinions from a group with diverse areas of academic focus and clinical expertise, gaps may remain. Additionally, evolving understanding of COVID-19 epidemiology might highlight additional factors not previously considered.

Despite these limitations, we present a collectively developed and iteratively refined list of data elements for observational research in COVID-19. By ensuring that long-term data collection efforts focused on COVID-19 include these candidate risk factors, mediators, and outcomes, studies leveraging the resulting datasets will be better positioned to yield accurate, informative, and actionable results.

## Acknowledgments

The investigators would like to acknowledge the contributions of Anna Barker and John Donnelly, who helped lead focus groups for causal diagram development. Each gave their permission to the authors for this Acknowledgments.

## Author contributions

**Conceptualization:** Andrew J. Admon, Pandora L. Wander, Amy S.B. Bohnert, Ann M. O’Hare, Matthew L. Maciejewski.

**Data curation:** Amy S.B. Bohnert, Matthew L. Maciejewski.

**Formal analysis:** Andrew J. Admon, Pandora L. Wander.

**Funding acquisition:** Theodore J. Iwashyna, George N. Ioannou, Edward J. Boyko, Denise M. Hynes, C. Barrett Bowling, Amy S.B. Bohnert, Ann M. O’Hare, Matthew L. Maciejewski.

**Investigation:** Andrew J. Admon, Edward J. Boyko, Matthew L. Maciejewski.

**Methodology:** Pandora L. Wander, Amy S.B. Bohnert, Valerie A. Smith, John Pura, Paul L. Hebert, Edwin S. Wong, Meike Niederhausen.

**Project administration:** Andrew J. Admon, Pandora L. Wander.

**Visualization:** Pandora L. Wander.

**Writing – original draft:** Andrew J. Admon, Matthew L. Maciejewski.

**Writing – review & editing:** Andrew J. Admon, Pandora L. Wander, Theodore J. Iwashyna, George N. Ioannou, Edward J. Boyko, Denise M. Hynes, C. Barrett Bowling, Amy S.B. Bohnert, Ann M. O’Hare, Valerie A. Smith, John Pura, Paul L. Hebert, Edwin S. Wong, Meike Niederhausen, Matthew L. Maciejewski.

## Supplementary Material
