# Supplementary material for: Consensus elements for observational research on COVID-19-related long-term outcomes
Source: Medicine (Baltimore). 2022 Nov 18;101(46):e31248. doi: 10.1097/MD.0000000000031248 (PMC9678399; doi:10.1097/MD.0000000000031248)
Supplement: Supplementary file 1 [file medi-101-e31248-s001.pdf]

Supplemental Content
Table 1. Expert Group Members list

Census Region	Terminal Degree	Focus area
East	MPH	Clinical research
East	MD, PhD	Critical illness epidemiology and health services research
East	PhD	Medical sociology; critical illness survivorship
East	MD	Racial and ethnic disparities; hewalth policy
Midwest	MD	Acute care epidemiology and health services research
Midwest	PhD	Chronic disease epidemiology and health services research
Midwest	MPH	Clinical research and implementation science
Midwest	MD	Critical care epidemiology and health disparities
Midwest	MD	Diabetes mellitus epidemiology and health services research
Midwest	MD, PhD	Epidemiology of dementia and chronic diseases
Midwest	MD	Health disparities; patient-provider communication
Midwest	MD, PhD	Infectious disease epidemiology
Midwest	PhD	Mental health epidemiology and health services research
Midwest	PhD	Observational research
Midwest	PhD	Sepsis epidemiology and implementation science
Midwest	MD	Sepsis epidemiology and survivorship
Non-US (Canada)	MD	critical care, clinical trials, and health services reesarch
South	PhD	Biostatistics
South	DrPH	Biostatistics; observational study design; causal inference
South	PhD	Observational research, health economics, and health policy
South	PhD	Population health, implementation science, health behavior
West	PhD	Biostatistics
West	PhD	Biostatistics, epidemiology
West	MS	Biostatistics; health services research
West	PhD	Biostatistics; observational research and clinical trials
West	MD	Cardiovascular disease epidemiology and treatment
West	PhD	Chronic disease epidemiology
West	MD	Chronic disease management; behavioral economics
West	MD	Chronic kidney disease epidmeiology and health services research
West	MD	Ciritcal illness epidemiology; palliative care
West	PhD	Clinical psychology; health disparities for vulnerable populations
West	PhD	Clinical psychology; palliative care; health care access
West	PhD	Epidemiological methods
West	MD	Epidemiology of chronic lung disease
West	MD	Epidemiology of diabetes mellitus
West	MD	Epidemiology of lung disease and pulmonary infections
West	MD	Epidemiology of non-communicable diseases; health services research
West	MD	Gastrointestinal and liver disease epidemiology; health services research
West	MD	Gastrointestinal disease epidemiology and health services research
West	MD, PhD	Health care delivery and quality
West	PhD, RN	Health care quality, access, and costs
West	PhD	Health economics and health care policy
West	MA	Health services and clinical research
West	PhD	Health services research, mental and behavioral health
West	MD	Infectious disease clinical care and epidemiology
West	MD, MSCI	Infectious disease, hospital care
West	MD	Inflammatory disease epidemiology and clinical care
West	MD	Lung disease epidemiology and treatment
West	MD	Medical informatics, medical decision making
West	PhD	Sociology; care teams; health services research
