# Supplementary material for: Consensus elements for observational research on COVID-19-related long-term outcomes
Source: Medicine (Baltimore). 2022 Nov 18;101(46):e31248. doi: 10.1097/MD.0000000000031248 (PMC9678399; doi:10.1097/MD.0000000000031248)
Supplement: Supplementary file 2 [file medi-101-e31248-s002.pdf]

Supplemental Content
Text 1. DAG Code

```
dag {
bb="0,0,1,1"
"12-month mortality" [outcome,pos="0.918,0.437"]
"Access to health care" [pos="0.126,0.545"]
"Appearance in VA Dataset" [adjusted,pos="0.855,0.742"]
"Comorbidities/Multimorbidity" [pos="0.175,0.462"]
"Health Related Knowledge" [pos="0.079,0.399"]
"Health-related behaviors" [pos="0.147,0.907"]
"Male sex at birth" [pos="0.637,0.886"]
"Nursing home residence" [pos="0.520,0.237"]
"Perceived Risk of Complications of COVID" [pos="0.167,0.047"]
"Perceived Risk of Contracting COVID" [pos="0.099,0.127"]
"SARS-CoV-2 Infection" [latent,pos="0.537,0.948"]
"SARS-CoV-2 Positivity" [exposure,pos="0.671,0.368"]
"Subjected to Racism" [pos="0.343,0.673"]
"Support Network Robustness" [pos="0.476,0.839"]
"Willingness/ability to get tested" [pos="0.295,0.155"]
"ZIP code (geographic location)" [pos="0.392,0.054"]
Age [pos="0.655,0.209"]
Frailty [pos="0.786,0.180"]
Occupation [pos="0.228,0.309"]
Wealth [pos="0.223,0.805"]
"Access to health care" -> "12-month mortality"
"Access to health care" -> "Appearance in VA Dataset"
"Access to health care" -> "SARS-CoV-2 Positivity"
"Comorbidities/Multimorbidity" -> "12-month mortality"
"Comorbidities/Multimorbidity" -> "Appearance in VA Dataset"
"Comorbidities/Multimorbidity" -> "Health-related behaviors"
"Comorbidities/Multimorbidity" -> "Perceived Risk of Complications of COVID"
"Comorbidities/Multimorbidity" -> "Perceived Risk of Contracting COVID"
"Comorbidities/Multimorbidity" -> "SARS-CoV-2 Infection"
"Comorbidities/Multimorbidity" -> "SARS-CoV-2 Positivity"
"Comorbidities/Multimorbidity" -> Frailty
"Health Related Knowledge" -> "12-month mortality"
"Health Related Knowledge" -> "Appearance in VA Dataset"
"Health Related Knowledge" -> "Health-related behaviors"
"Health Related Knowledge" -> "Perceived Risk of Complications of COVID"
```

"Health Related Knowledge" -> "Perceived Risk of Contracting COVID"

"Health Related Knowledge" -> "SARS-CoV-2 Positivity"

"Health Related Knowledge" -> "Willingness/ability to get tested"

"Health-related behaviors" -> "12-month mortality"

"Health-related behaviors" -> "Appearance in VA Dataset"

"Health-related behaviors" -> "SARS-CoV-2 Infection"

"Male sex at birth" -> "12-month mortality"

"Male sex at birth" -> "SARS-CoV-2 Infection"

"Male sex at birth" -> "SARS-CoV-2 Positivity"

"Nursing home residence" -> "12-month mortality"

"Nursing home residence" -> "SARS-CoV-2 Infection"

"Nursing home residence" -> "SARS-CoV-2 Positivity"

"Perceived Risk of Complications of COVID" -> "SARS-CoV-2 Infection"

"Perceived Risk of Complications of COVID" -> "Willingness/ability to get tested"

"Perceived Risk of Contracting COVID" -> "SARS-CoV-2 Infection"

"Perceived Risk of Contracting COVID" -> "Willingness/ability to get tested"

"SARS-CoV-2 Infection" -> "SARS-CoV-2 Positivity"

"SARS-CoV-2 Positivity" -> "12-month mortality"

"Subjected to Racism" -> "12-month mortality"

"Subjected to Racism" -> "Access to health care"

"Subjected to Racism" -> "Appearance in VA Dataset"

"Subjected to Racism" -> "Comorbidities/Multimorbidity"

"Subjected to Racism" -> "Health Related Knowledge"

"Subjected to Racism" -> "SARS-CoV-2 Positivity"

"Subjected to Racism" -> "Willingness/ability to get tested"

"Subjected to Racism" -> "ZIP code (geographic location)"

"Subjected to Racism" -> Occupation

"Support Network Robustness" -> "12-month mortality"

"Support Network Robustness" -> "Access to health care"

"Support Network Robustness" -> "Appearance in VA Dataset"

"Support Network Robustness" -> "SARS-CoV-2 Infection"

"Support Network Robustness" -> "Willingness/ability to get tested"

"Willingness/ability to get tested" -> "SARS-CoV-2 Positivity"

"ZIP code (geographic location)" -> "12-month mortality"

"ZIP code (geographic location)" -> "Appearance in VA Dataset"

"ZIP code (geographic location)" -> "SARS-CoV-2 Infection"

"ZIP code (geographic location)" -> "SARS-CoV-2 Positivity"

Age -> "12-month mortality"

```
Age -> "Comorbidities/Multimorbidity"
Age -> "Nursing home residence"
Age -> "SARS-CoV-2 Infection"
Age -> "SARS-CoV-2 Positivity"
Age -> "Willingness/ability to get tested"
Age -> Frailty
Frailty -> "12-month mortality"
Frailty -> "Nursing home residence"
Occupation -> "Appearance in VA Dataset"
Occupation -> "SARS-CoV-2 Infection"
Occupation -> "SARS-CoV-2 Positivity"
Occupation -> Wealth
Wealth -> "12-month mortality"
Wealth -> "Access to health care"
Wealth -> "Appearance in VA Dataset"
Wealth -> "Support Network Robustness"
Wealth -> "Willingness/ability to get tested"
Wealth -> "ZIP code (geographic location)"
}
```

Generated using Dagitty software, Johannes Textor, Benito van der Zander, Mark K. Gilthorpe, Maciej Liskiewicz, George T.H. Ellison. Robust causal inference using directed acyclic graphs: the R package 'dagitty'. International Journal of Epidemiology 45(6):1887-1894, 2016.
