# Supplementary material for: Consensus elements for observational research on COVID-19-related long-term outcomes
Source: Medicine (Baltimore). 2022 Nov 18;101(46):e31248. doi: 10.1097/MD.0000000000031248 (PMC9678399; doi:10.1097/MD.0000000000031248)
Supplement: Supplementary file 3 [file medi-101-e31248-s003.pdf]

**Supplemental Content**
**Text 2: Minimally sufficient adjustment sets.** Items appearing in bold are common to each of the two sets.

1. **Access to health care, Age, Appearance in VA Dataset, Comorbidities/Multimorbidity*, Health Related Knowledge, Health-related behaviors, Male sex at birth, Nursing home residence,** Occupation, Perceived Risk of Complications of COVID, Perceived Risk of Contracting COVID, **Subjected to Racism, Support Network Robustness,** Willingness/ability to get tested, **ZIP code (geographic location)**
2. **Access to health care, Age, Appearance in VA Dataset, Comorbidities/Multimorbidity*, Health Related Knowledge, Health-related behaviors, Male sex at birth, Nursing home residence, Subjected to Racism, Support Network Robustness,** Wealth, **ZIP code (geographic location)**

**Comorbidities include those plausibly affecting rates of SARS-COV-2 infection, test positivity, and mortality.*
